# Pathogenic *Leptospira* Secreted Proteases Target the Membrane Attack Complex: A Potential Role for Thermolysin in Complement Inhibition

**DOI:** 10.3389/fmicb.2017.00958

**Published:** 2017-05-30

**Authors:** Thais A. Amamura, Tatiana R. Fraga, Sílvio A. Vasconcellos, Angela S. Barbosa, Lourdes Isaac

**Affiliations:** ^1^Laboratory of Complement, Department of Immunology, Institute of Biomedical Sciences, University of São PauloSão Paulo, Brazil; ^2^Faculty of Veterinary Medicine, University of São PauloSão Paulo, Brazil; ^3^Laboratory of Bacteriology, Butantan InstituteSão Paulo, Brazil

**Keywords:** *Leptospira*, immune evasion, proteases, MAC, complement system

## Abstract

Leptospirosis is a zoonosis caused by spirochetes from the genus *Leptospira*. This disease is common in tropical and subtropical areas, constituting a serious public health problem. Pathogenic *Leptospira* have the ability to escape the human Complement System, being able to survive when in contact with normal human serum. In a previous study, our group demonstrated that supernatants of pathogenic *Leptospira* (SPL) inhibit the three activation pathways of the Complement System. This inhibition can be directly correlated with the activity of secreted proteases, which cleave the Complement molecules C3, Factor B (Alternative Pathway), C4 and C2 (Classical and Lectin Pathways). In this work, we analyze the activity of the leptospiral proteases on the components of Terminal Pathway of Complement, called the membrane attack complex (MAC). We observed that proteases present in SPL from different *Leptospira* strains were able to cleave the purified proteins C5, C6, C7, C8, and C9, while culture supernatant from non-pathogenic *Leptospira* strains (SNPL) had no significant proteolytic activity on these substrates. The cleavages occurred in a time-dependent and specificity manner. No cleavage was observed when we used whole serum as a source of C5–C9 proteins, probably because of the abundant presence of plasma protease inhibitors such as α_2_-macroglobulin. Complement protein cleavage by SPL was inhibited by 1,10-phenanthroline, indicating the involvement of metalloproteases. Furthermore, 1,10-phenanthroline- treated normal human serum diminished pathogenic leptospira survival. We also analyzed the proteolytic activity of thermolysin (LIC13322) a metalloprotease expressed exclusively by pathogenic *Leptospira* strains. Recombinant thermolysin was capable of cleaving the component C6, either purified or as part of the SC5b-9 complex. Furthermore, we found that the MAC proteins C6–C9 interact with thermolysin, indicating that this metalloprotease may have an additional inhibitory effect on these molecules by direct interactions. Finally, a functional assay demonstrated that thermolysin was able to inhibit MAC-dependent erythrocytes lysis. We conclude that proteases secreted exclusively by pathogenic *Leptospira* strains are capable of degrading several Complement effector molecules, representing potential targets for the development of new therapies and prophylactic approaches in leptospirosis.

## Introduction

Leptospirosis is considered the most important zoonosis worldwide, affecting approximately one million patients every year, being fatal in approximately 5–10% of cases (Costa et al., 2015). This disease is considered a global public health problem affecting especially developing countries with tropical or subtropical temperatures and deficient sewage conditions. This bacterial infection is caused by spirochetes from the genus *Leptospira* which includes pathogenic, intermediate and saprophytic (non-pathogenic) species (Ko et al., 2009; Fouts et al., 2016). In most cases, the infection is caused through contact with water or soil contaminated with leptospires shed in the urine of infected mammalian hosts, mainly rodents in urban areas.

The host immune responses against *Leptospira* are mostly dependent on phagocytosis, production of specific antibodies and activation of the Complement System. Once activated, several important biological functions are generated from this system contributing for *Leptospira* killing, such as production of opsonins, which facilitates the phagocytosis, increase in the production of specific antibodies and formation of the membrane attack complex (MAC) (reviewed in Fraga et al., 2011).

Membrane attack complex formation is initiated after the cleavage of the component C5 into two fragments: C5a and C5b. The latter fragment binds to the component C6 and exhibits a binding site for the component C7 forming the intermediary complex C5b67. C5b67 binds to the bacterial surface and now accepts the binding of C8, forming the complex C5b678. Several molecules of C9 are incorporated to this complex creating a channel through the membrane (C5b6789_n_). This channel is a lytic pore leading to flow of water and ions which culminates in osmotic unbalance and the cell rupture (Morgan et al., 2016). When in solution, the C5b67 complex binds to the regulatory S-protein (also called Vitronectin) forming the complex (SC5b67). S-Protein is a multi-functional glycoprotein of 75 kDa and plays a crucial role in many biological processes including cell migration, adhesion and inhibition of the MAC and coagulation. Even though this complex cannot bind to the cell membrane, it does not interfere with the binding of C8 and C9 to form SC5b6789_n_ (Tegla et al., 2011). Purified SC5b67 has been used to study the protein-protein interactions of these proteins (Müller-Eberhard, 1986; Bhakdi et al., 1988).

Several studies have investigated the immune evasion mechanisms employed by pathogenic *Leptospira* (reviewed in Fraga et al., 2016): (i) acquisition of host Complement regulatory proteins such as Factor H (Meri et al., 2005; Barbosa et al., 2009; Castiblanco-Valencia et al., 2012); C4b binding protein (Barbosa et al., 2009, 2010; Breda et al., 2015) and Vitronectin (da Silva et al., 2015); (ii) acquisition of host protease plasminogen/plasmin which cleaves C3b and C5 (Castiblanco-Valencia et al., 2016; and, iii) release of leptospiral proteases which cleave the central Complement component C3 and its fragments C3b and iC3b; Factor B; C4b and C2; inhibiting all three Classical, Alternative and Lectin Pathways of the Complement System (Fraga et al., 2014). All these proteolytic properties are exclusively observed in the supernatant from pathogenic *Leptospira* cultures (SPL) but not in the supernatant from saprophytic *Leptospira biflexa* strains cultures (SNPL), which explains their susceptibility to Complement killing (Fraga et al., 2014, 2016).

Several examples of pathogen-derived metalloproteases with the capacity to cleave Complement proteins have been described by different groups: elastase and alkaline protease from *Pseudomonas aeruginosa* (Hong and Ghebrehiwet, 1992), gelatinase from *Enterococcus faecalis* (Park et al., 2008) and aureolysin from *Staphylococcus aureus* (Laarman et al., 2011). Metalloproteases are the main class of proteases present in SPL capable of cleaving several Complement proteins since this activity is abolished by the treatment with 1,10-phenanthroline (Fraga et al., 2014). Among this class of proteases, the family of thermolysins is present exclusively in pathogenic *Leptospira* and is coded by four independent genes and one recombinant thermolysin has been shown to be able to cleave both purified C3 or C3 in whole normal human serum (Fraga et al., 2014).

Up to now, MAC degradation by proteases secreted by pathogenic *Leptospira* species, including thermolysins, has not been reported in the literature. Since MAC is important for the killing of several pathogens, we decided to investigate in this study if these pathogenic leptospiral proteases could degrade C5, C6, C7, C8, and C9.

## Materials and Methods

### Proteins, Antibodies, and Sera

The Complement proteins purified from human plasma C5, C6, C7, C8, C9 (C6–C9) and S-protein bound to C5b-9 (SC5b-9 complex), C5b6, and specific goat anti-human polyclonal antibodies were purchased from Complement Technology. Secondary anti-goat IgG antibody conjugated with peroxidase was purchased from KPL. Normal human serum (NHS) was obtained from healthy volunteers, after informed and signed consent (CEPSH #1206).

### *Leptospira* Strains and Culture Supernatants

We employed two saprophytic (non-pathogenic) *Leptospira* strains: *L. biflexa* serovar Andamana strain CH11 and *L. biflexa* serovar Patoc strain Patoc I, and five pathogenic strains: *L. interrogans* serovar Pomona strain Pomona, *L. interrogans* serovar Kennewiki strain Fromm, *L. interrogans* serovar Copenhageni strain 10A, *L. interrogans* serovar Cynopteri strain 3522C and *L. interrogans* serovar Panama strain CZ 214. All *Leptospira* strains were cultivated for 7 days in modified Ellinghausen-McCullough-Johnson-Harris (EMJH) medium at 29°C under aerobic conditions (Barbosa et al., 2006). The supernatants containing the secreted proteases were obtained as described before by Fraga et al. (2014). Briefly, fresh bacterial suspensions containing 1.0 × 10^9^ leptospires were incubated at 37°C for 4 h. The supernatants were collected after centrifugation, passed through a 0.22-mm filter, aliquoted and immediately frozen at -80°C until use.

### Expression and Purification of *Leptospira interrogans* Thermolysin

The recombinant PepSY-M4 fragment from thermolysin (IUBMB EC 3.4.24.27) contains the prodomain PepSY and the catalytic domains Peptidase_M4 and Peptidase_M4_C (amino acids 144–794) as described by Fraga et al. (2014). *Escherichia coli* DH5a and *E. coli* BL21 (SI) were used for cloning and expression, respectively.

### Quantification of Protein Concentration and Electrophoresis of Leptospiral Supernatants

Total SPL or SNPL protein concentrations were determined using Pierce BCA Kit. Before proteins were separated by 8–15% SDS-PAGE (Laemmli, 1970) they were solubilized in reducing loading buffer, heated for 3 min at 96°C. Both SPL and SNPL were considered free of lipopolysaccharide (LPS) according to the Limulus Amebocyte Lysate test (<0.125 endotoxins units/ml).

### Proteolytic Activity and Inhibition Assays

Supernatants of pathogenic *Leptospira*, SNPL (1.5 μg of total secreted proteins) or recombinant thermolysin (0.5–6 μg) were incubated with purified human C6, C7, C8, or C9 proteins (0.25 μg each) in a final volume of 25 μl (ratio: 5 supernatant proteins/1 Complement protein), up to 4 h at 37°C. We also used 10% NHS as a source of Complement proteins (in 22 μl of final volume). To evaluate the proteolytic specificity of SPL, proportionally lower concentrations of SPL proteins (0.07, 0.45, or 0.9 μg of total protein) were added to 5 μg of each C6–C9 proteins (respectively, ratios 0.01:1, 0.04:1, and 0.018/1 of supernatant protein/complement protein) diluted in PBS pH 7.4 in a final volume of 17 μl. When we assayed the proteolytic specificity of SPL to cleave purified C5, 0.06, 0.3, and, 0.6 μg of SPL were incubated with 3 μg of purified C5 in a final volume of 17 μl for 1 h or 4 h at 37°C.

We also evaluated the SPL cleavages of C6–C9 present in the SC5b-9 complex incubating 5 μg of this complex with 0.9 μg of SPL, for 4 h at 37°C (17 μl of final volume). In some experiments, SPL was pre-incubated with inhibitors of serine-, metallo-, cysteine-, or aspartyl-proteases [5 mM phenylmethylsulfonyl fluoride (PMSF); 5 mM 1,10-phenanthroline; 28 μM E-64 or 5 μM pepstatin, respectively] or 3 μM human plasma protease inhibitor α_2_-macroglobulin (α_2_M) (Roche) for 30 min before adding purified C6–C9 proteins. The cleavage products were subjected to 15% SDS-PAGE and analyzed by Western blot, as described by Fraga et al. (2014). After transferring proteins to nitrocellulose membranes, non-specific protein interactions were blocked with 10% skimmed milk diluted in PBS containing 0.05% Tween (PBS-T), followed by incubation with polyclonal goat anti-human C5–C9 antibodies, washed with PBS-T and incubated with rabbit peroxidase-conjugated anti-goat IgG antibodies. Positive signals were detected by enhanced chemiluminescence (West Pico, Pierce). When indicated, cleavage products were visualized after electrophoresis and staining with Coomassie Blue (Sigma–Aldrich). Molecular weight standards (Page Ruler) were purchased from Fermentas.

### Ligand Affinity Blotting

The interaction between thermolysin and C6 was analyzed by ligand affinity blotting (overlay) and ELISA. In the first case, recombinant thermolysin and a negative control (BSA) were subjected to 12% SDS–PAGE under non-reducing conditions and transferred to nitrocellulose membranes (Castiblanco-Valencia et al., 2012). After blocking with 10% skimmed milk in PBS-T, C6 (10 μg/ml) was added to the membrane for 90 min at 37°C. After washing, bound proteins were detected with goat anti-human C6 polyclonal antibody (1:5000), followed by incubation with peroxidase-conjugated secondary anti-goat antibody (1:5000). Positive signals were detected by enhanced chemiluminescence (West Pico, Pierce). The interactions between recombinant thermolysin and C7, C8, and C9 were also investigated by ELISA, following the same protocol. The interaction between Complement proteins (C6, C7, C8, and C9) and thermolysin was quantified by ELISA. Microtiter plates were coated with thermolysin or BSA (negative control) (100 μl; 10 μg/ml) for 16 h at 4°C. The wells were washed with PBS-T and blocked with PBS containing 3% BSA for 2 h at 37°C. After this period, Complement proteins (C6, C7, C8, or C9) were added (100 μl; 0–5 μg/ml) to the plate. The reaction was incubated for 1 h at 37°C and then washed with PBS-T. Bound proteins were detected with goat anti-human C6, anti-C7, anti-C8, or anti-C9 polyclonal antibodies (1:5000), followed by peroxidase-conjugated secondary anti-goat antibody (1:5000). As substrate, we used o-phenylenediamine dihydrochloride (Pierce) and the absorbance was measured at 492 nm. The dissociation constant (K_d_) of the interactions was calculated by non-linear regression by fitting the data to the equation Y = B_max_^∗^X/(K_d_ + X) using GraphPad Prism 5.0 (GraphPad Software, Inc.). To test if the interaction of thermolysin with C6 is dependent on ionic strength or is affected by heparin, similar ELISA protocol was performed immobilizing thermolysin on microtiter wells (100 μl; 10 μg/ml) and then adding a fixed amount of C6 (1 μg) in the presence of increasing NaCl concentrations (0 to 600 mM, diluted in 10 mM Na_2_HPO_4_ and 1.8 mM KH_2_PO_4_) or heparin (0.05 to 1 μg, diluted in PBS), respectively.

### Hemolytic Assay

To investigate if recombinant thermolysin would interfere in MAC-mediated erythrocyte lysis, we used a hemolytic assay previously described by Hallström et al. (2009) and da Silva et al. (2015). 2 × 10^8^ sheep erythrocytes/ml in Veronal-Buffered Saline were pre-incubated with purified C5b6 (2 μg/ml) in a final volume of 100 μl for 1 h at room temperature. LIC10301 is an outer membrane lipoprotein identified in pathogenic *L. interrogans* serovar Copenhageni, as predicted by the PSORT program^1^ (Nakai and Kanehisa, 1991). LIC10301 does not bind Factor H or C4BP (Castiblanco-Valencia et al., 2012; da Silva et al., 2015). In different fresh tubes, leptospiral recombinant proteins, thermolysin (6.25 to 50 μg/ml) or LIC10301 (50 μg/ml, negative control) were pre-incubated with 2 μg/ml C7 for 30 min at 37°C. Subsequently, C8 (2 μg/ml) and C9 (0.2 μg/ml) proteins were added to the mixture for 15 min at 37°C. Next, C5b6-coated erythrocytes were added to the mixture of recombinant thermolysin-C7-C8-C9 or recombinant LIC10301-C7-C8-C9. After incubation for 30 min at 37°C, the erythrocyte suspensions were centrifuged and lysis was evaluated by measuring absorbance of the supernatants at 540 nm in the supernatants. The percentage of lysis was expressed considering 100% lysis when no leptospiral protein was added to the reaction. Statistical analyses were performed using ANOVA. Significant differences (^∗^) were considered when *p* ≤ 0.05.

## Results

### Proteases Secreted by Pathogenic *Leptospira* Cleave Purified Terminal Pathway Proteins

**FIGURE 1:**
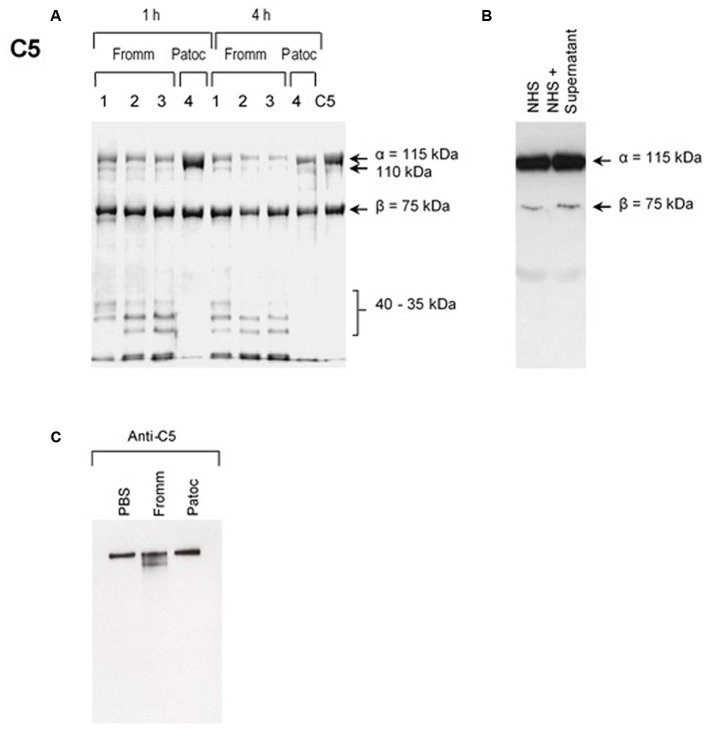
Supernatants of pathogenic (SPL) proteases cleave purified component C5 and C5b from the MAC. **(A)** Different concentrations (1) 0.6 μg; (2); 0.12 μg; (3) 0.06 μg of SPL from *Leptospira interrogans* serovar Kennewicki strain Fromm or (4) 0.06 μg SNPL from *L. interrogans* serovar Patoc strain Patoc I were incubated with 3 μg of purified C5 (left) for 1 or 4 h at 37°C. **(B)** Alternatively, SPL was incubated with 10% normal human serum (NHS) for 1 h at 37°C (right). The cleavage products were separated after SDS-PAGE and analyzed by Western blot using polyclonal anti-human C5. **(C)** SPL (Fromm) and SNPL (Patoc) were incubated with 5 μg of purified SC5b-9 complex for 4 h at 37°C. The cleavage product was analyzed by under non-reducing conditions by Western blot using polyclonal anti-C5 antibody.

Our first approach was to investigate if SPL or SNPL could cleave purified component C5. As shown in the **Figures 1A–C**, C5-cleavage products were observed exclusively when we used SPL from *L. interrogans* serovar Kennewicki strain Fromm. Proteases present in the SPL cleaved C5 α chain, generating 35–40 kDa fragments even when using reduced amounts (0.6–0.06 μg) of SPL proteases to 3 μg C5 in 17 μl (**Figure 1A**). In addition, C5b as part of SC5b-9 complex was cleaved in the presence of SPL (**Figure 1C**). However, C5 cleavage was not observed when whole NHS was used as a source of this protein (**Figure 1B**).

Since C6, C7, C8, and C9 share common protein domains (Hadders et al., 2012; Bubeck, 2014), we decided to explore the proteolytic activity of leptospiral culture supernatants on these proteins. For this, seven different strains of *Leptospira* were selected: five pathogenic and two saprophytes (non-pathogenic). SPL or SNPL were independently incubated with purified C6, C7, C8, or C9 proteins (ratio w/w: 5 of supernatant: 1 of Complement protein). The cleavages were analyzed by Western blot employing specific antibodies against each protein.

**FIGURE 2:**
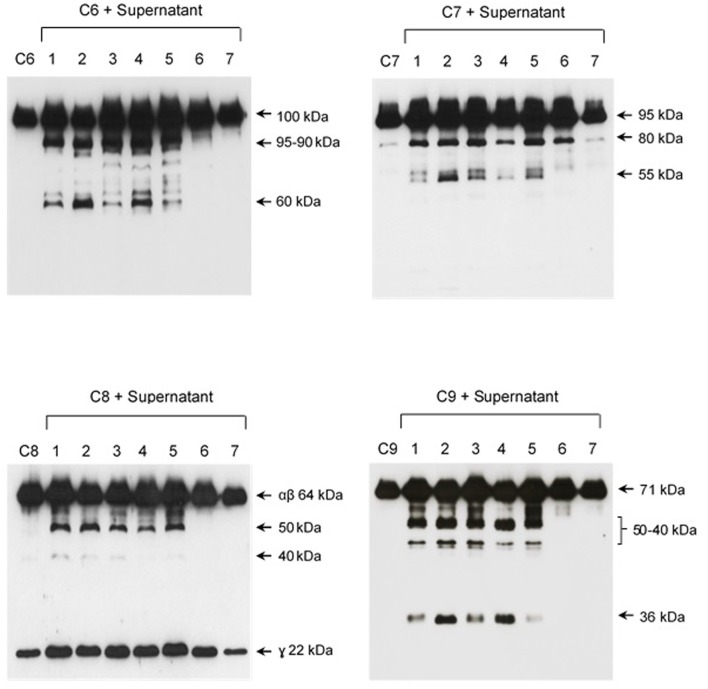
Proteases secreted by pathogenic *Leptospira* cleave complement proteins C6–C9. SPL (1.5 μg) from five different *Leptospira* strains (lanes 1–5) and SNPL (1.5 μg) from two different saprophytic *Leptospira* strains (lanes 6 and 7) were incubated with the purified Complement proteins C6, C7, C8, and C9 (0.25 μg each) for 1 h at 37°C. As a negative control, C6–C9 proteins were individually incubated with only PBS. *Leptospira* strains used: (1) *L. interrogans* serovar Pomona strain Pomona, (2) *L. interrogans* serovar Kennewiki strain Fromm, (3) *L. interrogans* serovar Copenhageni strain 10A, (4) *L. interrogans* serovar Cynopteri strain 3522C, (5) *L. interrogans* serovar Panama strain CZ 214), (6) *L. biflexa* serovar Andamana strain CH11 and (7) *L. biflexa* serovar Patoc strain Patoc I. The cleavages were analyzed by Western blot using specific polyclonal antibodies.

**FIGURE 3:**
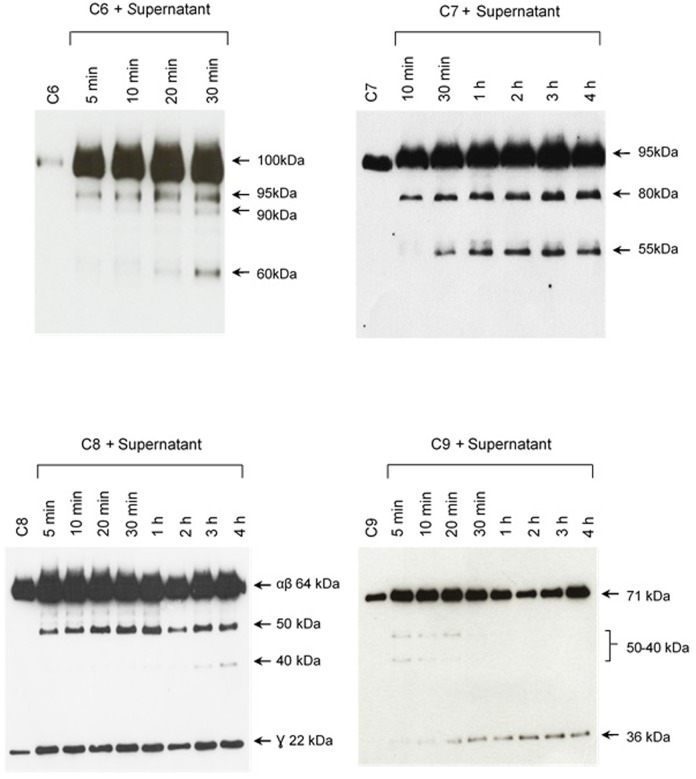
The cleavage of MAC proteins by leptospiral proteases is time-dependent. Pathogenic *L. interrogans* serovar Kennewiki strain Fromm was allowed to secrete proteases for 4 h at 37°C. The supernatant was collected and incubated with C6, C7, C8, or C9 for increasing times (5 min to 4 h) at 37°C. The cleavages were analyzed by Western blot with polyclonal antibodies against the Complement proteins.

**FIGURE 4:**
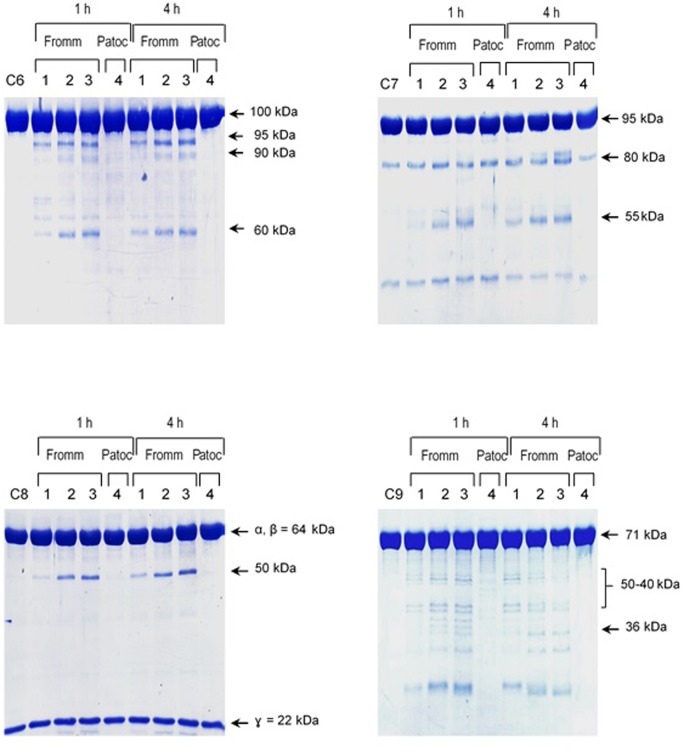
Reduced amounts of leptospiral proteases are able to cleave components of the Membrane Attack Complex. Pathogenic *L. interrogans* serovar Kennewicki strain Fromm and saprophytic *L. biflexa* serovar Patoc strain Patoc I (1.0 × 10^9^ bacteria) were allowed to secrete proteases for 4 h at 37°C. Different quantities of the supernatants, (1) 0.07 μg, (2) 0.4 μg, (3 and 4) 0.9 μg were incubated with 5 μg of the Complement proteins C6, C7, C8, or C9 (corresponding to 1/70; 1:15 and 1:5 w/w supernatant/Complement protein), for 1 h and 4 h at 37°C. The cleavages were analyzed by SDS-PAGE stained with Coomassie blue.

**FIGURE 5:**
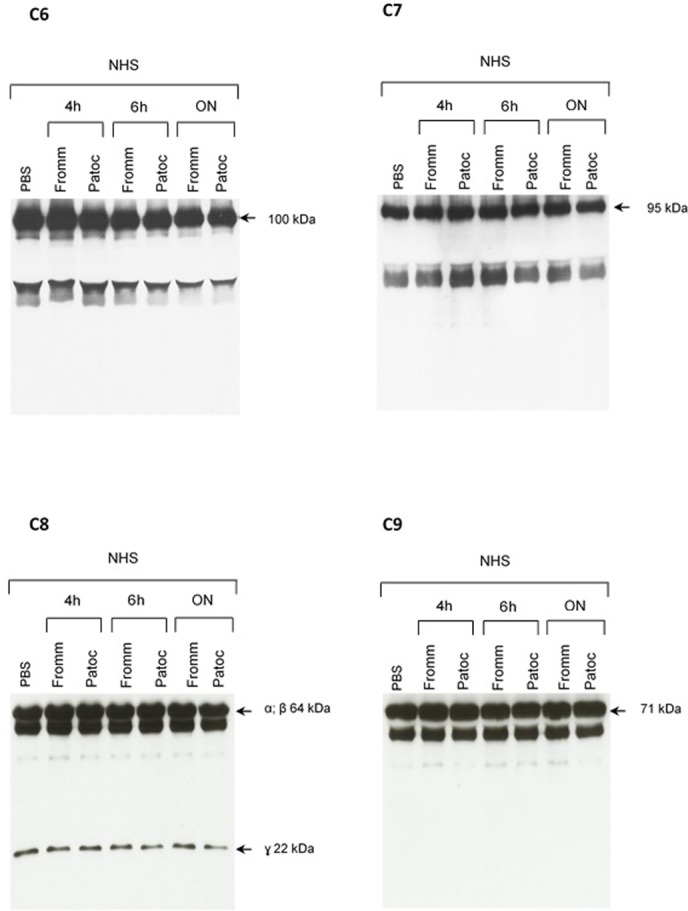
Proteolytic activity of SPL or SNPL on C6-C9 proteins present in normal human serum. SPF from *L. interrogans* serovar Kennewicki strain Fromm and SNPL from *L. biflexa* serovar Patoc strain Patoc I were incubated with normal human serum (as a source of C6–C9 proteins; NHS) for 4 h, 6 h or overnight (ON). Normal human serum diluted only in PBS was used as a negative control. The cleavage fragments were analyzed by Western blot using specific polyclonal antibodies.

The pattern of cleavage of C6–C9 proteins by SPL was similar for all five different strains of pathogenic leptospires employed in this study. On the other hand, no significant proteolytic activity on these purified Complement proteins was observed using SNPL from the two *L. biflexa* strains (**Figure 2**). The C6 polypeptide chain (∼100 kDa) was degraded by SPL producing mainly 95–90 kDa and 60 kDa fragments. The component C7 (95 kDa single chain) was also cleaved by SPL generating ∼80 and 55 kDa fragments. Intact C8 protein is composed of three polypeptide chains: α and β chains (both ∼64 kDa) and γ chain (∼22 kDa). SPL cleavage of C8 generated 50 and 40 kDa bands. The component C9 is composed of a single 71 kDa polypeptide chain and after incubation with SPL 50–40 kDa and ∼36 kDa fragments were observed by Western blotting analysis. In these assays, C6–C9 protein degradation by SPL (1.5 μg) was time-dependent and cleavage products could be observed before 30 min of incubation (**Figure 3**). The proteolytic activity specificity of SPL was observed even when relatively small amounts were used in relation to the concentrations of purified C6–C9 proteins as indicated in **Figure 4**, the cleavage products were still observed even when we used a SPL: Complement proteins mass ratio of 0.015. However, no C6–C9 cleavage products were observed when we treated NHS with SPL (**Figure 5**).

### Inhibition of Proteolytic Activity

**FIGURE 6:**
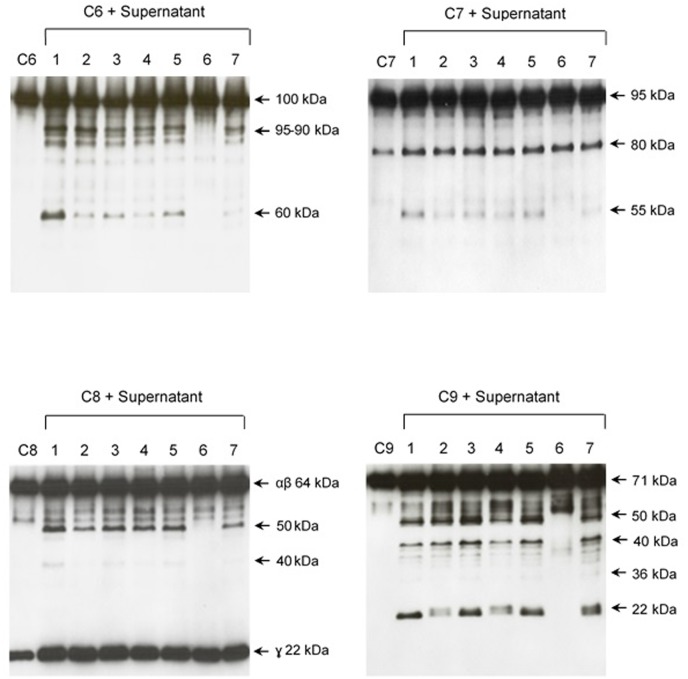
The protease activity of SPL is inhibited by 1.10-phenanthroline. SPL from *L. interrogans* serovar Kennewiki strain Fromm culture was previously treated with proteases inhibitors of serine-proteases (PMSF; lane 4), cysteine-proteases (E-64; lane 5), metalloproteases (1.10-phenanthroline; lane 6) or aspartyl-proteases (pepstatin; lane 7) before adding Complement proteins. The vehicles of the protease’s inhibitors were used as controls (lane 1) PBS, (lane 2) ethanol: H_2_O (1:1), (lane 3) ethanol. The cleavages of C6, C7, C8, and C9 were analyzed by Western blot with specific polyclonal antibodies.

**FIGURE 7:**
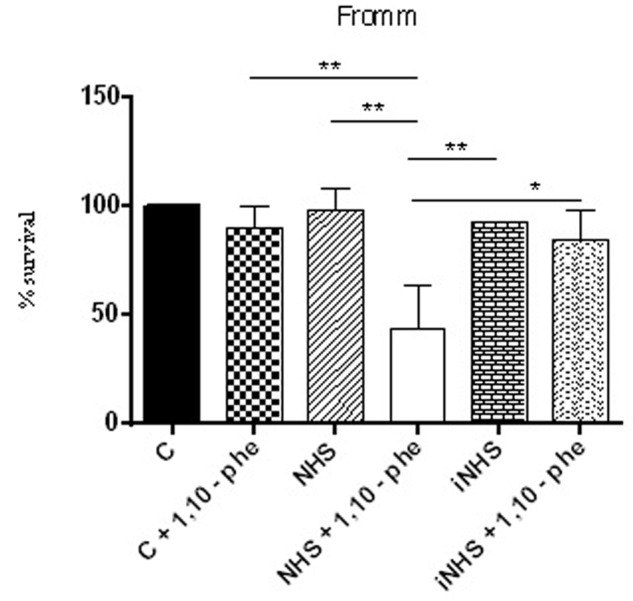
Inhibition of metalloproteases by 1,10-phenanthroline reduces pathogenic leptospire survival in the serum. Suspensions of *L. interrogans* serovar Kennewicki strain Fromm was incubated with EMJH containing 10% BSA (C; negative control); 40% normal human serum (NHS) or heat inactivated serum (iNHS) in the presence or absence of 5 mM 1,10-phenanthroline. The number of viable leptospires observed in the negative control (C) was considered 100 % survival. Statistical analyses were performed using ANOVA. ^∗^*p* ≤ 0.05, ^∗∗^*p* ≤ 0.001.

To identify which classes of proteases present in SPL are involved in the cleavage of purified C6–C9 proteins, we employed the following inhibitors: PMSF (inhibits serine proteases), E-64 (inhibits cysteine proteases), 1,10-phenanthroline (inhibits metalloproteases) and pepstatin (inhibits aspartyl proteases). Only 1,10-phenanthroline was able to inhibit the cleavage of the above Complement proteins by proteases present in supernatant from pathogenic *L. interrogans* serovar Kennewicki strain Fromm (**Figure 6**). This result strongly suggests that the metalloproteases represent one of the major classes of proteases present in the SPL involved in the cleavage of C6–C9 purified proteins. As shown in **Figure 7** pathogenic *L. interrogans* serovar Kennewicki strain Fromm is resistant to NHS. However, bacterial survival diminishes by at least 50% in the presence of 1,10-phenanthroline-treated NHS which confirms the importance of secreted leptospiral metalloproteases for evasion from Complement activation.

**FIGURE 8:**
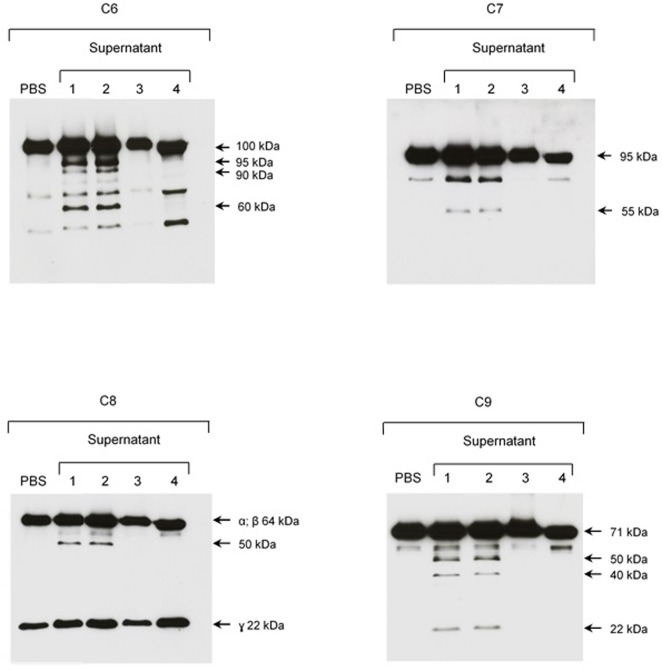
The plasma protease inhibitor α2M inhibits cleavage of purified C6–C9 proteins by SPL proteases. SPF from *L. interrogans* serovar Kennewicki strain Fromm was incubated with (1) PBS; (2) Ethanol; (3) 1,10-phenanthroline; and, (4) α2M for 30 min at room temperature. Next, C6–C9 purified proteins were added to reaction, incubating for 2 h at 37°C. The cleavage fragments were analyzed by Western blot using specific polyclonal antibodies.

Since no cleavage of C6-C9 proteins was observed when we incubated SPL with NHS, we investigated if α_2_M, an abundant plasma protease inhibitor, would have the same effect than 1,10-phenanthroline. **Figure 8** shows that when we pre-incubated SPL with α_2_M before adding purified C6–C9 proteins, cleavage was also inhibited.

### SPL and Recombinant Thermolysin Cleave C6 in the SC5b-9 Complex

**FIGURE 9:**
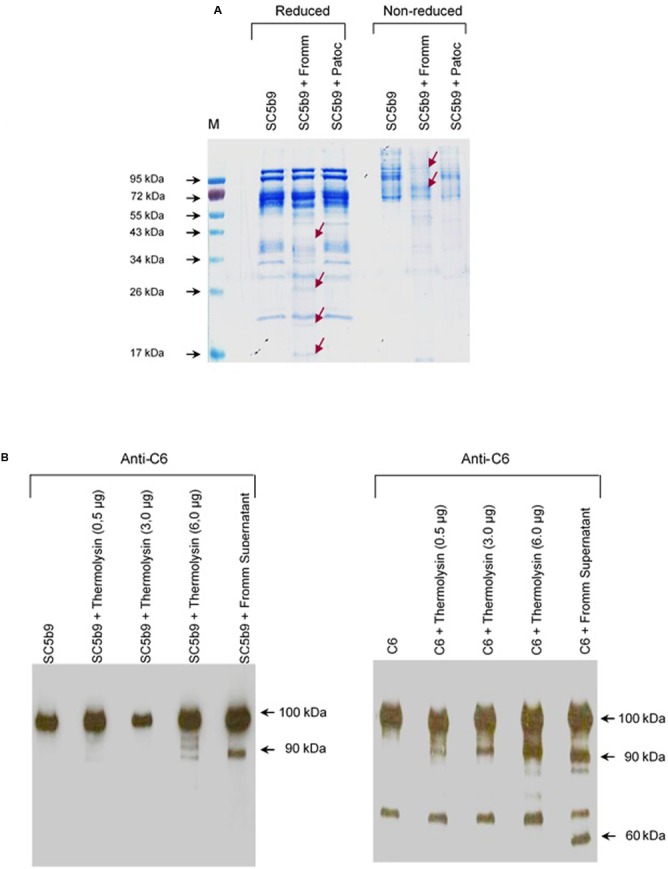
Leptospiral proteases and recombinant Thermolysin target the component C6 from the Membrane Attack Complex. **(A)** The soluble complex SC5b-9 was incubated with the supernatants of saprophytic *L. biflexa* serovar Patoc strain Patoc I or pathogenic *L. interrogans* serovar Kennewiki strain Fromm, for 2 h at 37°C. The cleavages were analyzed by SDS-PAGE stained with Coomassie blue. (M) Molecular weight marker (Page Ruler, Fermentas). **(B)** Recombinant Thermolysin had their proteolytic activity tested. The purified component C6 or SC5b-9 were incubated with increasing amounts of Thermolysin (0.5–6 μg) for 4 h at 37°C. SPL from *L. interrogans* serovar Kennewiki strain Fromm was also incubated with SC5b-9 in the same conditions. The cleavages were analyzed by Western blot with anti-human C6 polyclonal antibody.

Next, we decided to investigate the proteolytic activity of the SPL proteases on the C6–C9 assembled in the MAC. Since most of circulating MAC complexes rapidly binds to the S-protein (also known as Vitronectin), SPL or SNPL were incubated with SC5b-9 complex for 2 h at 37°C under reducing and non-reducing conditions (**Figure 9A**). Again, only SPL presented proteolytic activity on the SC5b-9 complex. Under reducing conditions, we observed degradation generating products with 43–34 kDa proteins, and the concomitant generation of cleavage fragments sized between 26 and 17 kDa. Under non-reducing conditions, a marked degradation of proteins between 95 and 72 kDa was observed followed by several fragments with lower molecular mass. Different than what was described with individual C6–C9 proteins (**Figure 2**), SPL proteases were able to cleave C6 but not C7, C8, or C9 when they are organized in the MAC (data not shown).

Next, investigated if a recombinant *Leptospira* thermolysin previously showed be present in the SPL (Fraga et al., 2014) would cleave C6–C9 individual proteins or complexed in SC5b-9. Recombinant thermolysin (LIC13322, fragment PepSY M4) was incubated separately with each of purified C6, C7, C8, and C9 proteins or with the soluble SC5b9 complex and the cleavage products were analyzed by Western blot employing specific antibodies. In this assay, we observed that only C6 was cleaved by recombinant thermolysin LIC13322 (**Figure 9B**). No cleavage fragments were observed when C7, C8, or C9 were employed (data not shown). The recombinant thermolysin cleaved both purified C6 and as an integral part of the soluble SC5b9 complex. The cleavage of C6 by thermolysin has a similar pattern to that seen with it purified when we used SPL with a common 90 kDa degradation product. Still, the C6 cleavage was more pronounced in the presence of SPL when compared in the presence of recombinant thermolysin (60 kDa additional band) (**Figure 9B**).

**FIGURE 10:**
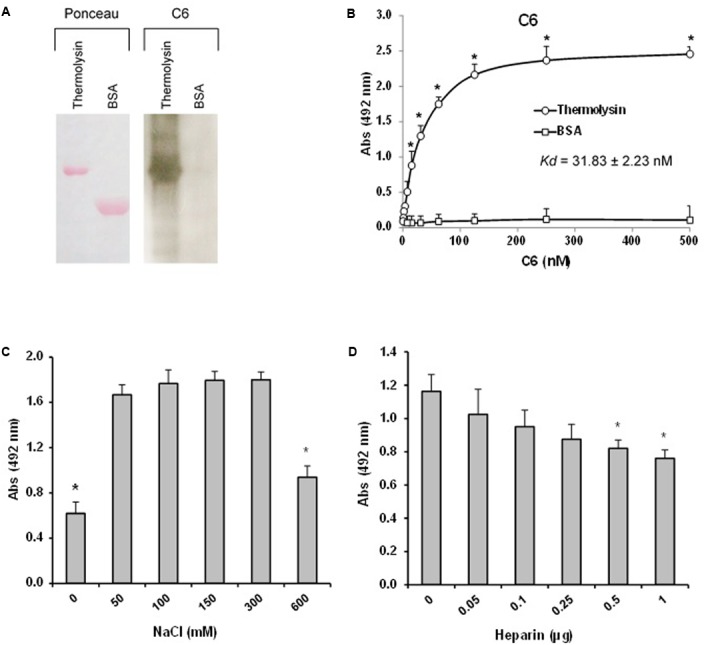
The leptospiral thermolysin interacts with C6. **(A)** Affinity blotting. (Left) Recombinant Thermolysin and BSA were subjected to 12% SDS–PAGE under non-reducing conditions, transferred to a nitrocellulose membrane, and stained with Ponceau S. (Right) The membrane containing the immobilized proteins was incubated with purified C6, and binding was detected with anti-human C6 polyclonal antibody. **(B)** Microtiter plates were coated with Thermolysin (1 μg) and different amounts of C6 were added. Binding was detected with anti-human C6 polyclonal antibody. BSA was included as a negative control. Each point represents the mean absorbance value at 492 nm ± SD of three independent experiments, each performed in duplicate. Binding of C6 to Thermolysin was compared with the binding of this molecules to BSA by the two-tailed *t*-test (^∗^*p* < 0.05). The dissociation constant (*K*_d_) of the interactions was calculated by fitting the data to the equation Y = B_max_^∗^X/(K_d_ + X) using GraphPad Prism 5.0 (GraphPad Software, Inc.). **(C)** Interaction of Thermolysin with C6 is dependent on ionic strength. C6 (1 μg) diluted in different NaCl concentrations (0–600 mM) were added to Thermolysin immobilized on microtiter wells. Binding was detected with anti-human C6 polyclonal antibody. **(D)** Interaction of thermolysin with C6 is affected by heparin. C6 (1 μg) diluted in different heparin solutions (0–1 μg) were added to thermolysin immobilized on microtiter wells. Each point represents the mean absorbance value at 492 nm ± SD of three independent experiments, each performed in duplicate. Data were analyzed using ANOVA test (^∗^*p* < 0.05).

The interaction between thermolysin and C6 was further explored by Far-western blot (*overlay*) in which recombinant thermolysin or BSA (negative control) were subjected to 12% SDS-PAGE and transferred to a nitrocellulose membrane. This membrane containing the immobilized proteins was incubated with purified C6 and the interaction was analyzed using anti-C6 specific antibody. **Figure 10A** shows that C6 interacts with immobilized recombinant thermolysin. We confirmed the interaction between thermolysin and C6 by ELISA, and the dissociation constant of the complex (*K*_d_) was estimated to be of 31.8 ± 2.2 nM (**Figure 10B**).

We then investigated whether the thermolysin – C6 interaction is dependent on ionic strength. Since heparin affects MAC assembly (Sahu and Pangburn, 1993), we decided to investigate the effect of the addition of heparin on the thermolysin–C6 interaction. Thermolysin was immobilized and C6 protein was added in the presence of increasing concentrations of NaCl (0–600 mM; **Figure 10C**) or different amounts of heparin (0.05 to 1 μg; **Figure 10D**). We observed that the interaction between C6 and thermolysin was reduced only at high concentrations of NaCl (600 mM). Furthermore, we observed a dose-dependent inhibition by heparin (0.5 to 1.0 μg), suggesting that thermolysin may interact at least partially with C6 through heparin binding sites.

### Recombinant Thermolysin Inhibits MAC-Mediated Hemolysis

**FIGURE 11:**
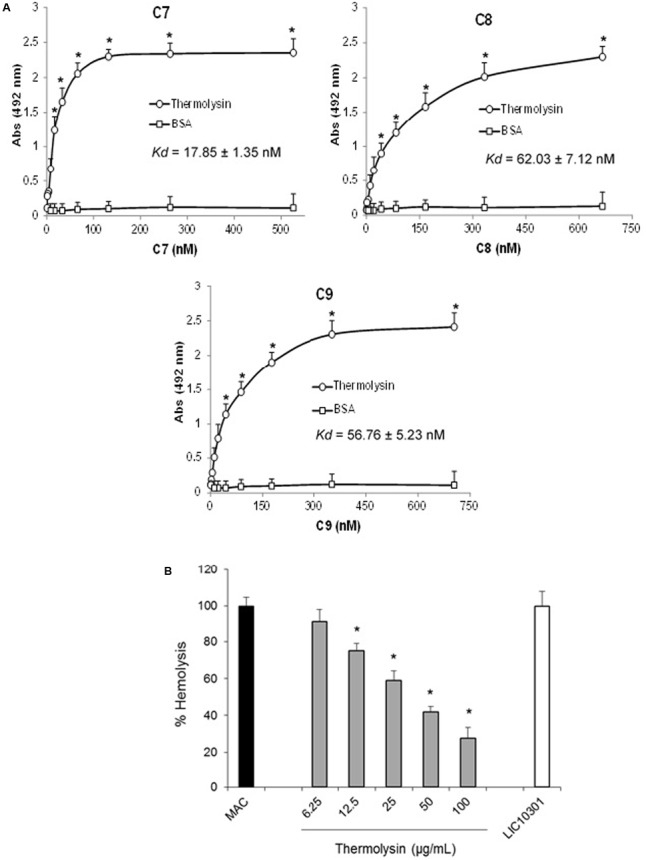
Thermolysin inhibits MAC-mediated hemolysis. **(A)** Microtiter plates were coated with thermolysin (1 μg) and different amounts of C7, C8, or C9 were added. Binding was detected with specific antibodies against the complement proteins. BSA was included as a negative control. Each point represents the mean absorbance value at 492 nm ± SD of three independent experiments, each performed in duplicate. Binding of MAC proteins to thermolysin was compared with the binding of these molecules to BSA by the two-tailed *t*-test (^∗^*P* < 0.05). The dissociation constant (*K*_d_) of the interactions was calculated by fitting the data to the equation Y = B_max_^∗^X/(K_d_ + X) using GraphPad Prism 5.0 (GraphPad Software, Inc.). **(B)** Thermolysin (6.25–50 μg/ml) or LIC10301 (50 μg/ml) were pre-incubated with C7, C8, and C9 and then added to C5b6-coated sheep erythrocytes. After incubation, cell lysis was measured, and the lysis in the absence of inhibitor (MAC) was set to 100%. Statistical analyses were performed using ANOVA. ^∗^*p* ≤ 0.05.

Several protein domains are common among C6–C9 proteins. We therefore asked if recombinant thermolysin, although it does not exert proteolytic activity on C7, C8, and C9, could interact directly with these molecules resulting in an additional inhibition effect by direct interaction, facilitating *Leptospira* Complement evasion. The interaction between thermolysin and the C7, C8, and C9 components of the MAC was analyzed by ELISA (**Figure 11A**). Recombinant thermolysin was immobilized and different amounts of the C6–C9 proteins were added separately to enable the calculation of the dissociation constant (*K*_d_). We could indeed observe interactions between the *Leptospira* protease and the Complement C7, C8, and C9 components, with *K*_d_ values of 17.8 ± 1.3 nM; 62.0 ± 7.1 nM; and 56.8 ± 5.2 nM, respectively (**Figure 11A**). The interactions between thermolysin and C6–C9 components inhibits MAC-mediated hemolysis (**Figure 11B**) in a dose-dependent manner. These results demonstrated that the proteases secreted by the pathogenic *Leptospira* may inhibit the formation of MAC by cleaving C6, and/or by direct binding to C6-C9.

## Discussion

Pathogenic *Leptospira* have acquired several mechanisms to escape from Complement System activation, one of the main participants of the innate and acquired immunity against leptospirosis (Fraga et al., 2016). Recently, we demonstrated that pathogenic but not saprophytic *Leptospira* secrete metalloproteases able to cleave key Complement proteins. This allows these bacteria to avoid the three Complement activation pathways (Fraga et al., 2014).

Extracellular metalloproteases are generally well-conserved and ubiquitous among bacteria, playing important roles in both pathogenic and non-pathogenic microorganisms. Immune evasion is among the roles played by these proteolytic enzymes. In *E. faecalis*, the extracellular gelatinase GelE was shown to cleave C3 and iC3b leading to the consumption of C3 and a decrease in phagocytosis (Park et al., 2008). *Clostridium perfringens* lambda-toxin, a thermolysin-like metalloprotease, was shown to degrade extracellular matrix and plasma proteins, including C3. Injection of lambda-toxin in dorsal skin of mice increased vascular permeability and caused haemorrhagic edema (Jin et al., 1996). In spirochetes, a surface-exposed metalloprotease named pallilysin was shown to contribute to *Treponema pallidum* dissemination by degrading fibrin clots (Houston et al., 2012).

In this study, we demonstrated that SPL and recombinant thermolysin (LIC13322) cleave individual purified C5–C9 proteins. In addition, C5b and C6 are also cleaved by these proteases when they are part of the SC5b6789_n_ complex. In addition, the interaction between C6 and recombinant thermolysin was dependent on ionic strength and heparin concentration.

Even though recombinant thermolysin (LIC13322) did not cleave C7, C8, or C9 proteins, this metalloprotease bound directly to these components and inhibited MAC-mediated hemolytic activity. To our knowledge, this is the first study that shows that leptospiral secreted proteases contribute to evasion of host immune response by degrading proteins from the terminal pathway or by binding directly to them.

Membrane attack complex directly lyses a wide range of microbes, including a wide range of Gram-negative bacteria. Unexpectedly, only very few studies have explored pathogens’ strategies to escape from MAC-mediated lysis. Fernie-King et al. (2001) described a streptococcal inhibitor of complement that prevents MAC formation because it interferes with C5b-C7 and C5b-C8 complexes. In the spirochete *Borrelia burgdorferi*, a membrane-bound protein that blocks the MAC formation by binding to C8 and C9 was described by Pausa et al. (2003). The helminth *Schistosoma mansoni* expresses a surface protein that binds to the C8β subunit and consequently inhibits MAC formation (Parizade et al., 1994). Recently, da Silva et al. (2015) demonstrated that pathogenic *Leptospira* but not saprophytic ones bind to the host regulatory protein Vitronectin, controlling in this way the complete and functional MAC formation. One study (Honda-Ogawa et al., 2013) demonstrated that a cysteine protease from *Streptococcus pyogenes* (SpeB) was able to cleave several Complement proteins including C6–C9. The *S. pyogenes speB* mutant had a more intense C9 deposition on its surface and a lower survival capacity when in the presence of normal human serum.

We observed that SPL and thermolysin (LIC13322) were able to cleave purified C5–C9 but not when normal human serum was employed as a source of Complement proteins. This observation is particularly relevant during the acute phase of this infection when *Leptospira* is circulating in the blood (Levett, 2015). However, once leptospires reach other sites such as liver, kidney, or lung the local protein–protein interactions may be different than those in the circulation. To study protein-protein interactions in the serum is not trivial, since there is a relative high viscosity and total protein concentration (70–100 mg/l) and they are competing among themselves even with low affinity. Another important consideration is that plasma proteases inhibitors such as anti-trypsin and α2M are commonly present in the circulation. The inhibitor α2M (720 kDa) is present in human plasma at concentration of 4.4 ± 0.2 mg/ml, which corresponds to 6.9 ± 0.4% of total plasma protein (Thieme et al., 2015). It is considered an inflammatory acute phase protein in rodents but not in humans (Isaac et al., 1999). The best known function of α2M is its ability to covalently trap a large spectrum of proteases, including trypsin, chymotrypsin, elastase, or metalloproteinases (Sottrup-Jensen, 1989). Here we observed that the cleavage of C6–C9 proteins by SPL is inhibited by the presence of α2M which could explain, at least in part, why when we used normal human serum, the cleavage of C6–C9 proteins by SPL was not observed. One could speculate that during the incubation period (2–30 days) after pathogenic *Leptospira* have invaded an injured skin or mucosa, the secretion of leptospiral proteases could be locally important to avoid MAC deposition on the bacterial surface. Consequently, MAC-mediated lysis would be diminished, favoring the bacterial dissemination in the host. The role of these secreted proteases during leptospirosis using *in vivo* experimental models remains to be further investigated.

All four C6–C9 proteins share common domains: thrombospondin type I repeats, lipoprotein receptor class A repeat, membrane attack complex/perforin and epidermal growth factor like repeat. In addition, C6 and C7 share more two types of domains: complement control protein repeats and factor I/membrane attack complex 6/7 modules (Hadders et al., 2012; Bubeck, 2014). Considering these structural similarities and the fact that thermolysin binds to C6, we speculated that thermolysin could bind directly to C7, C8, and C9, even without any proteolytic activity. Confirmation of this hypothesis brings up to the possibility that some leptospiral products would interfere in the full Complement activation just by binding to the MAC proteins.

Our results suggest that thermolysin (LIC13322) and possibly other proteases secreted by pathogenic *Leptospira*, may interfere with the formation and deposition of MAC on the bacterial surface by two non-exclusive mechanisms: (i) cleavage of C5 on its own or of C5b as part of the SC5b-9 complex (ii) cleavage of C6 on its own or as part of the SC5b-9 complex; and (iii) direct protein–protein interaction between thermolysin and C6–C9 individual proteins. In this way, we believe that proteases secreted exclusively by pathogenic *Leptospira* can contribute to its evasion from Complement System activation by avoiding MAC formation and pathogen lysis. This knowledge may help us to design better vaccines in the future and to better control this zoonosis.

## Ethics Statement

This study was carried out in accordance with the recommendations of Comissão de Ética em Pesquisa com Seres Humanos (CEPSH#1206) with written informed consent from all subjects.

## Author Contributions

TA, TF, and LI designed the experiments. TA and TF performed the experiments. LI, TA, TF, and AB wrote the manuscript, SV provided the bacterial cultures and all authors have read the manuscript.

## Conflict of Interest Statement

The authors declare that the research was conducted in the absence of any commercial or financial relationships that could be construed as a potential conflict of interest.

## Footnotes

1 http://psort.nibb.ac.jp
